# Effects of porous structure and oxygen functionalities on electrochemical synthesis of hydrogen peroxide on ordered mesoporous carbon

**DOI:** 10.1038/s42004-024-01194-3

**Published:** 2024-05-13

**Authors:** Abdalazeez Ismail Mohamed Albashir, Xingyu Lu, Xueya Dai, Wei Qi

**Affiliations:** 1 School of Materials Science and Engineering, University of Science and Technology of China, Shenyang, 110016 Liaoning People’s Republic of China; 2 Shenyang National Laboratory for Materials Science, Institute of Metal Research, Chinese Academy of Sciences, Shenyang, Liaoning People’s Republic of China

**Keywords:** Electrocatalysis, Electrocatalysis, Electrochemistry, Porous materials

## Abstract

Two-electron oxygen reduction reaction (2e^−^ ORR) is a promising alternative to energy-intensive anthraquinone process for hydrogen peroxide (H_2_O_2_) production. Metal-free nanocarbon materials have garnered intensive attention as highly prospective electrocatalysts for H_2_O_2_ production, and an in-depth understanding of their porous structure and active sites have become a critical scientific challenge. The present research investigates a range of porous carbon catalysts, including non-porous, microporous, and mesoporous structures, to elucidate the impacts of porous structures on 2e^−^ ORR activity. The results highlighted the superiority of mesoporous carbon over other porous materials, demonstrating remarkable H_2_O_2_ selectivity. Furthermore, integration of X-ray photoelectron spectroscopy (XPS) data analysis with electrochemical assessment results unravels the moderate surface oxygen content is the key to increase 2e^−^ ORR activity. These results not only highlight the intricate interplay between pore structure and oxygen content in determining catalytic selectivity, but also enable the design of carbon catalysts for specific electrochemical reactions.

## Abstract (web summary)

The anthraquinone process is currently the predominant method for large-scale H_2_O_2_ production, but the reaction’s high energy consumption and hazardous waste generation spur the search for alternatives. Here, the authors investigate the two-electron oxygen reduction reactivities of metal-free non-porous, micro-, and mesoporous carbon catalysts to elucidate the impacts of porous structures, finding mesoporous structures show the highest H_2_O_2_ production selectivity.

## Introduction

Hydrogen peroxide (H_2_O_2_) is a crucial and versatile chemical with wide-ranging applications, including chemical industry, environmental protection and medical field^1–3^. The anthraquinone oxidation/reduction process currently stands as the predominant method for large-scale H_2_O_2_ production^4^. However, despite its massive scale applications, the high energy consumption and hazardous waste generation make it non-environmentally friendly^5^. Consequently, there is an urgent need to develop efficient and environmentally friendly methods for H_2_O_2_ production, particularly for on-site applications. In this regard, electrochemical two-electron oxygen reduction reaction (2e^−^ ORR) has been considered as a potentially advantageous strategy for generating H_2_O_2_ due to its mild operation condition, green reactants such as air and water, and the capability of being powered by green electricity^6–8^.

Despite the advantages offered by the 2e^−^ ORR for H_2_O_2_ electrosynthesis, a significant challenge remains due to the lack of an efficient catalyst that could effectively enhance the selectivity towards H_2_O_2_. Recent investigations have demonstrated the 2e^−^ ORR activity of precious-metal electrocatalysts, such as Pt^9^ and Pd^10^, as well as transition metals like Co^11^, Fe^12^, Ni^13^ etc. However, their high cost and limited availability of resources hinder the extensive utilization in large-scale applications. Metal-free carbon nanomaterials have attracted significant attention as potential electrocatalysts for H_2_O_2_ production due to their sustainability, abundance, cost-effective, and tunable surface properties^14,15^. As pristine pure carbon catalysts typically exhibit poor catalytic performance, modification strategies such as porous construction^16^, defect engineering^17^ surface modification^18^, and heteroatom doping, including oxygen^19^, nitrogen^20^, fluorine^21^, sulfur^22^, phosphorous^23^, and boron^24^ have emerged as beneficial approaches to enhance the catalytic efficiency of carbon materials. These modifications not only improve catalyst efficiency but also maintain the principles of sustainability and cost-effective, which both crucial for advancing H_2_O_2_ electrosynthesis technologies.

Two critical factors have been found to significantly impact the 2e^−^ ORR catalytic activity of carbon materials, including porous structure and the oxygen functional groups^25,26^. First, well-ordered micro-/mesoporous structures provide a larger surface area for the reaction interface, more accessible active sites, and improved electron transfer paths during 2e^−^ ORR^27,28^. Assessing the impact of each pore size in porous carbon (microporous and mesoporous) is crucial in the electrochemical production of H_2_O_2_^29–32^. Related research has shown that mesoporous structures are preferable to microporous ones, as the former facilitates faster mass transfer and promotes the formation of H_2_O_2_. For instance, it has been reported that a mesoporous-dominant nitrogen-doped carbon material exhibited higher H_2_O_2_ selectivity than a microporous-dominant nitrogen-doped carbon^33^. However, the role of porous structure in enhancing H_2_O_2_ activity is debated, and it has also been reported that carbon materials with greater microporosity content demonstrated a relatively high H_2_O_2_ partial current due to the enhanced density of defect sites on the surface^32^. Consequently, by comprehensively assessing the effects of each pore size in porous materials, scientists can gain a deeper understanding of the fundamental principles governing the electrochemical synthesis of H_2_O_2_ and enhance the efficiency and selectivity of the overall process.

Oxygen functional groups also play critical roles in the 2e^−^ ORR process, highlighting their potential to fine-tune the electrocatalytic properties and kinetics of the H_2_O_2_ synthesis reaction^34,35^. Some early conclusive evidence has demonstrated that some oxygen functional groups, such as carboxyl (-COOH) and ether (-C-O-C) moieties, may serve as active sites in the 2e^−^ ORR process through experimental study or density functional theory (DFT) calculation methods^25^. Recent studies on successful functionalization of carbon nanotubes with specific oxygen groups highlighted the crucial role of the -C = O group in achieving excellent 2e^−^ ORR selectivity^36^. However, understanding the intricate interplay between oxygen content and catalytic behavior is crucial for rational designing effective and selective carbon catalysts for the 2e^−^ ORR process.

In this comprehensive study, we systematically assessed the influence of pore size and oxygen content on the catalytic activity of carbon materials for electrochemical synthesis of H_2_O_2_. Among the array of carbon catalysts examined, mesoporous carbon exhibits outstanding electrocatalytic activity, demonstrating significantly higher H_2_O_2_ selectivity up to 88% at 0.4 V_RHE_ in alkaline media (0.1 M KOH) compared to its other porous carbon counterparts. This elevated selectivity of mesoporous carbon can be attributed to its precisely tailored pore structure, which facilitated the mass transportation of reactants and products, coupled with efficient confinement of reaction intermediates, ultimately favoring the preferred formation of H_2_O_2_ over competing side reactions. In addition, analyses from X-ray photoelectron spectroscopy (XPS) and electrochemical activity assessments unveiled that the C = O may be the main active site of 2e^−^ ORR, and appropriate oxygen content could benefit the selective synthesis of H_2_O_2_. Furthermore, we explored the synergistic interplay between porous structure and oxygen species, and an optimized activity at 100% H_2_O_2_ selectivity highlighted the significant roles of both factors in determining the catalytic activity. This study serves as a foundational platform for comprehending the intricate relations governing catalyst activity, and it contributes not only to the fundamental understanding of structure-function correlations but also offers valuable insights for the development of improved electrocatalysts tailored for efficient and selective H_2_O_2_ electrosynthesis.

## Results and discussion

### Insights into effect of porous structure on catalytic activity

**Fig. 1:**
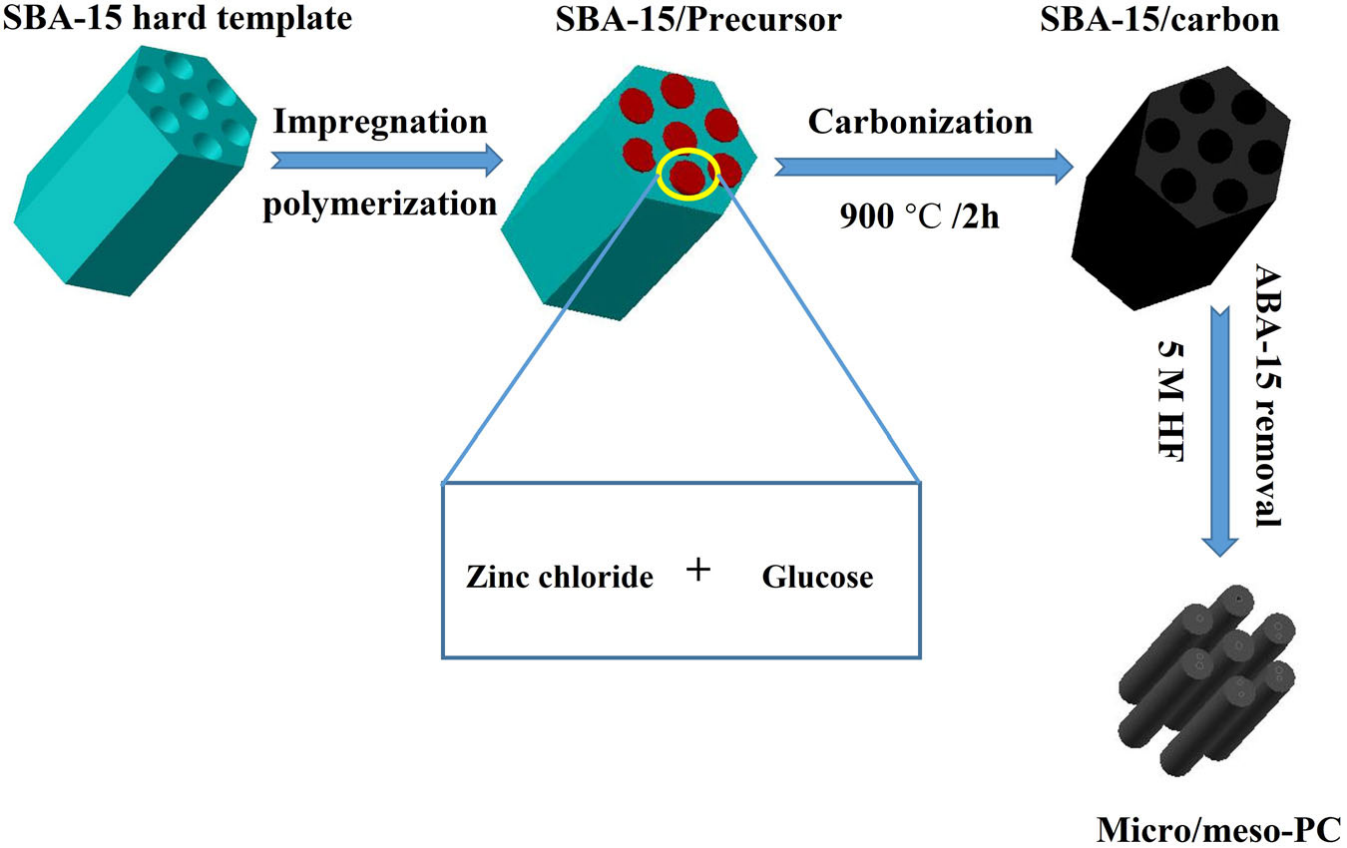
Schematic illustration of Micro/meso-PC synthesis process. The schematic diagram depicts the synthesis process of Micro/meso-PC, illustrating the key steps involved, impregnating, polymerization, carbonization, and template Removal.

To systematically explore the influence of porous structure on the efficiency of electrochemical synthesis of hydrogen peroxide, a series of porous carbon (PC) materials spanning a wide range of porosities, from micropores to macropores, were synthesized utilizing ZnCl_2_ salts, Y-zeolite (SiO_2_/Al_2_O_3_) and SBA-15 hard templates as pore-forming agents. In a typical procedure, the synthesis involved impregnating glucose and ZnCl_2_ into the pores of SBA-15 hard templates, followed by subsequent polymerization, carbonization, and template removal, as illustrated in Fig. 1. To precisely assess the impact of the porous structure on the 2e^−^ ORR performance, hierarchical porous carbon (Hiera-PC), microporous carbon (micro-PC), and non-porous carbon (template-free-C) were also fabricated for comparisons (the detailed fabrication process was provided in the Methods section).

**Fig. 2:**
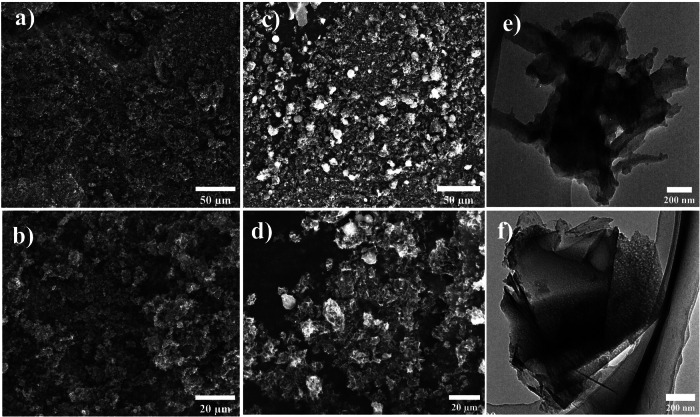
Morphology characterizations of meso-PC and micro-PC. **a**, **b** SEM and **c** TEM images of meso-PC. **d**, **e** SEM and **f** TEM images of micro-PC.

As illustrated in SEM images in Supplementary Fig. 1a, b, the Template-free-C exhibits a glassy-like structure, indicative of its limited porosity and low specific surface area. Upon impregnating glucose into the pores of Y-zeolite or SBA-15 templates, a significant increase in surface roughness could be observed in the Meso-PC (Fig. 2a, b) and micro-PC (Fig. 2c, d) catalysts, signifying a substantial increase in specific surface area. The SEM images of Hiera-PC (Supplementary Fig. 1c, d) and micro/meso-PC (Supplementary Fig. 1e, f) reveal irregular pore sizes attributable to the introducing of ZnCl_2_ as a pore-forming agent. The transmission electron microscopy (TEM) images show that the density of mesoporous channels in pure SBA-15 are evident (Supplementary Fig. 2a, b). These mesoporous channels are completely filled with the carbon precursors in the following impregnation procedure, resulting in mesoporous features within the carbon material after the template removal process (Fig. 2e)^37^. Furthermore, the micro-PC catalyst displays a significant number of microporous channels, as shown in Fig. 2f. X-ray diffraction (XRD) measurements (Supplementary Fig. 3) reveal two diffraction peaks centered at around 24° and 44°, corresponding to the (002) and (100) faces of hexagonal carbon^38^.

**Fig. 3:**
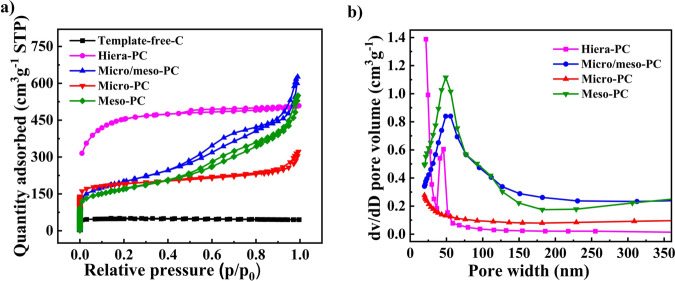
Pore characterizations of catalysts. **a** N_2_-adsorption-desorption isotherms, and **b** pore-size distributions of Template-free-C (Black), Hiera-PC (purpule), micro/meso-PC(Blue), micro-PC (Red) and Meso-PC (Green).

N_2_-adsorption-desorption isotherm measurements (Fig. 3a, b) reveal that the micro-PC catalyst exhibits an absence of hysteresis loops in the medium-pressure region (adsorption-desorption region for mesopores), indicating a higher volume of micropores. In contrast, the isotherms of meso-PC displayed large hysteresis loops in the medium-pressure region, signifying the presence of a higher volume content of mesopores. The absence of N_2_ uptake at both lower and higher-pressure regions for Template-free-C indicates its poor porous structure. Additionally, the increased loops observed in micro/meso-PC at higher pressure indicates greater microporous volume, which can be ascribed to the addition of ZnCl_2_. During carbonization process, ZnCl_2_ acts as a chemical activation agent, decomposing into volatile compounds under high temperatures^39^. This decomposition is critical to forming voids within the carbon matrix and leads to the emergence of a hierarchical pore structure, which includes both micro-and mesopores. The BET surface area of template-free-C, hiera-PC, micro-PC, micro/meso-C, and meso-PC catalysts are determined at 169, 1571, 858, 705, and 594 m^2^ g^−1^, respectively (Supplementary Table 1).

**Fig. 4:**
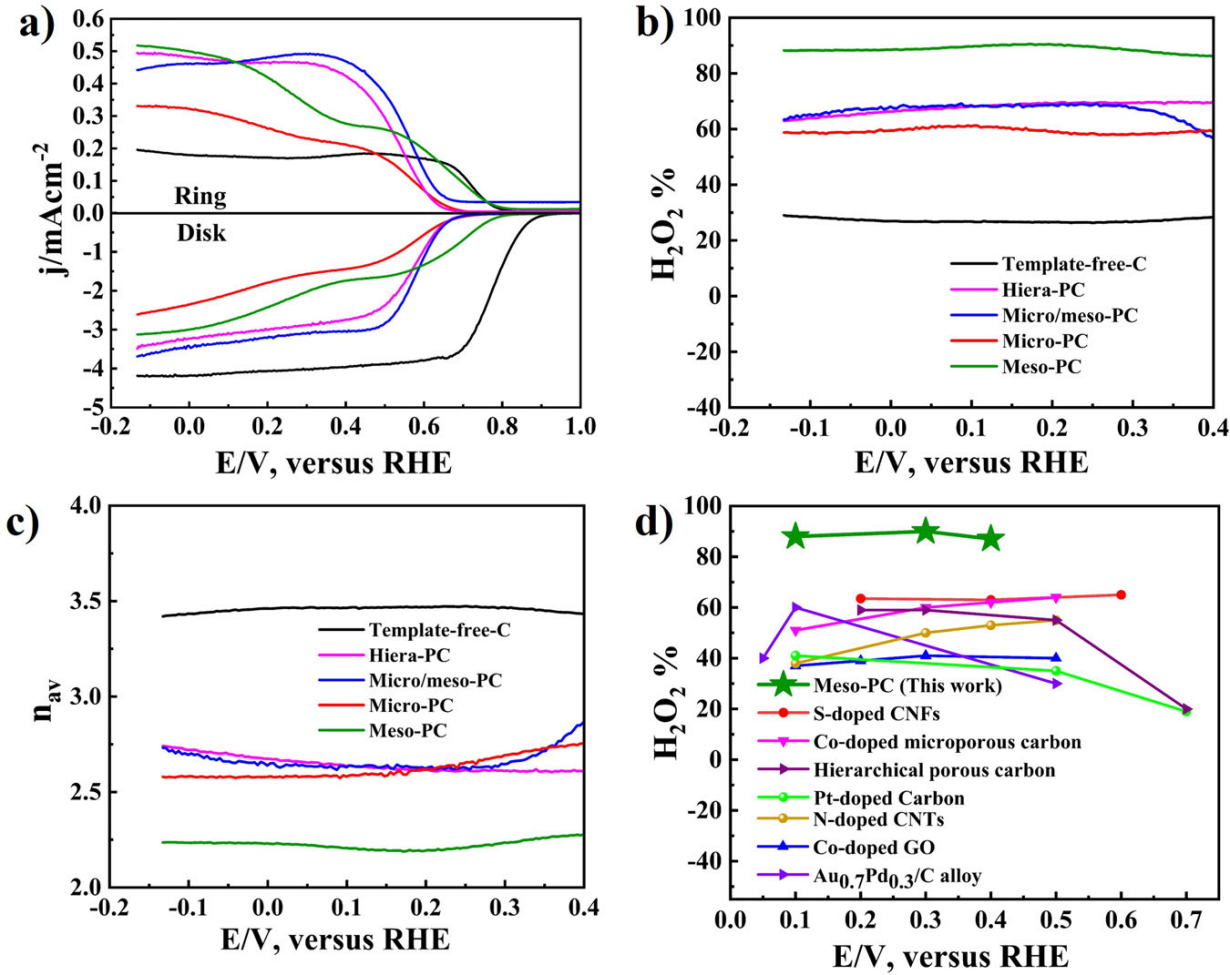
Electrochemical evaluation in rotating ring-disk electrode (RRDE). **a** SCV-RRDE profiles, **b** hydrogen peroxide selectivity (H_2_O_2_%), and **c** average number of electron transferred (*n*_av_) of template-free-C (Black), hiera-PC (purpule), micro/meso-PC(Blue), micro-PC (Red) and meso-PC (Green) in O_2_-saturated 0.1 M KOH solution, respectively. **d** H_2_O_2_% selectivity of Meso-PC comparing with previously reported carbon-based or metal-based electrocatalysts.

The electrochemical ORR process was evaluated using a rotating ring-disk electrode (RRDE) in an oxygen-saturated 0.1 M KOH electrolyte solution. From LSV curves it is observed that the Meso-PC exhibited a higher ring current density (*j*_D_) compared to all porous carbon and Template-free-C catalysts (Fig. 4a), indicating its high catalytic selectivity toward 2e^−^ ORR. The calculated values for the ring current density (*j*_R_) and disk current density (*j*_D_) for Template-free-C, Hiera-PC, micro-PC, micro/meso-PC, and Meso-PC were 0.12, 0.0.49, 0.0.44, 0.0.33 and 0.0.52 jmAcm^−2^, and −4.1, −3.4, −3.6, −2.59 and 3.09 jmAcm^−2^, respectively. The H_2_O_2_ selectivity for template-free-C, hiera-PC, micro-PC, micro/meso-PC and meso-PC were calculated at 28%, 69%, 58%, 60%, and 88%, respectively (Fig. 4b). Moreover, the transferred electron numbers for template-free-C, hiera-PC, micro-PC, micro/meso-PC and meso-PC are calculated to be 3.43, 2.60, 2.82, 2.80, and 2.24, respectively (Fig. 4c), suggesting that porous carbon catalysts exhibit higher 2e^−^ ORR selectivity than non-porous carbon. Notably, the meso-PC electrocatalyst exhibits superior catalytic activity and H_2_O_2_ selectivity, which is attributed to its improved mass transfer during the ORR process, facilitating the rapid release of generated H_2_O_2_ and preventing its complete 4e^−^ reduction to water^40^. The lower H_2_O_2_ selectivity of micro-PC should be attributed to its microporous structure, which confines H_2_O_2_ within small pores and leading to its further 4e^−^ reduction to H_2_O as the final product^30^.

Interestingly, we find that the specific surface area may not significantly affect H_2_O_2_ selectivity, as observed in the comparison between Hiera-PC (higher specific surface area of 1571 m^2^ g^−1^) and Meso-PC (lower surface area of 594 m^2^ g^−1^). While there is typically a correlation between specific surface area and catalytic activity because of the increased number of exposed active sites^41^, but it is not the only factor determining catalytic activity. The nature, distribution, and accessibility of active sites, along with the diffusion and confinement of species involved in the H_2_O_2_ synthesis within the porous structure, also play critical roles^33^. In this case, mesoporous carbon possesses a more favorable pore architecture for facilitating the necessary mass transportation and timely release of reaction intermediates, thereby enhancing H_2_O_2_ selectivity despite having a lower overall surface area compared with all other porous carbon catalysts (Supplementary Table 1). Compared with previously reported electrocatalysts, the H_2_O_2_ selectivity of the mesoporous carbon catalyst (meso-PC) is found to be significantly higher than most carbon-based materials and even comparable to some metal-based catalysts (Fig. 4d). To evaluate the 2e^−^ ORR performance across different pH conditions, all porous materials and Template-free-C, were also assessed in 0.1 M H_2_SO_4_ (Supplementary Fig. 4a–c). We observed a reduction in H_2_O_2_ selectivity in acidic media compared to alkaline conditions. This decrease can be attributed to a shift in the ORR pathway from 2e^−^ ORR to four-electron oxygen reduction reaction (4e^−^ ORR), resulting in H_2_O production. Typically, alkaline solutions facilitate the 2e^−^ ORR due to the presence of hydroxide ions (OH^−^), whereas acidic conditions are rich in protons (H^+^) tend to favor the four-electron oxygen reduction reaction (4e^−^ ORR) process. To enhance selectivity in acidic media, future work would focus on modifying the surface chemistry of carbon-based catalysts with specific functional groups or composite materials that leverage synergistic effects between carbon and other catalytically active sites optimized for acidic media.

### Insights into effect of oxygen functionalities on catalytic activity

**Fig. 5:**
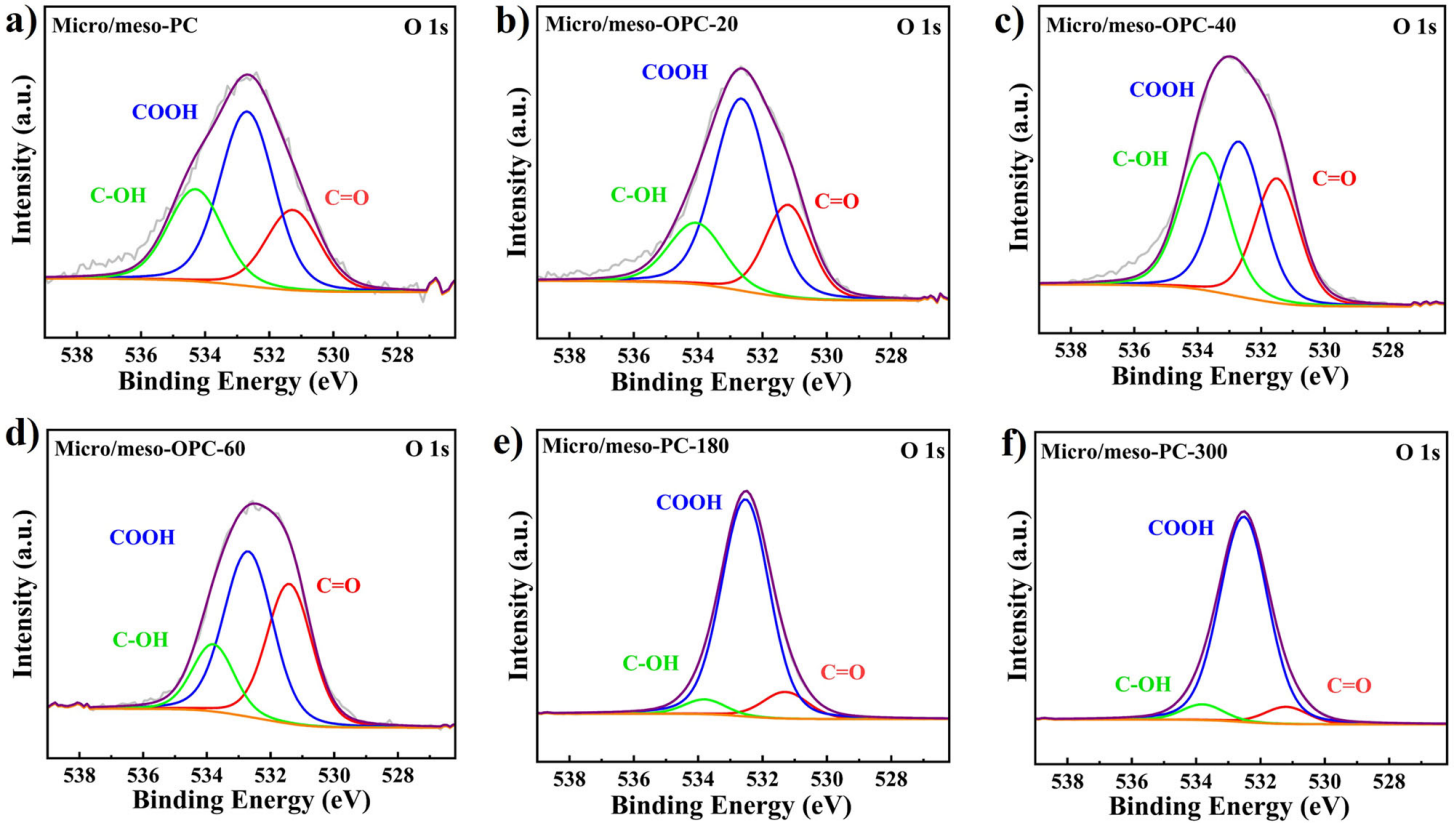
XPS Deconvolution of high-resolution O 1 s spectra (-C = O (carbonyl, Red), -COOH (carboxyl, Blue) and -C-OH (hydroxyl, Green)). **a** Micro/meso-PC, **b** micro/meso-OPC-20, **c** micro/meso-OPC-40, **d** micro/meso-OPC-60, **e** micro/meso-OPC-180 and **f** micro/meso-OPC-300, respectively.

To get an in-depth understanding on the oxygen species responsible for electrochemical H_2_O_2_ production, a series of oxidized samples with varying oxygen contents are synthesized undergoing nitric acid oxidation with varying duration time. The micro/meso-PC catalyst material is chosen as an ideal catalyst for identifying surface oxygen functional groups due to its intermediate selectivity and diverse pore sizes. The XPS survey spectra (Supplementary Fig. 5a) shows that micro/meso-OPC-x samples comprise carbon, nitrogen, and oxygen elements with slight silicon residue (0.37–0.4%) remaining in the catalysts structure after exclusive acid etching (Supplementary Fig. 5b). As indicated by the XPS results, the Zn element was completely evaporated during the carbonization process (Supplementary Fig. 5c), resulting in a porous structure in carbon material. The oxygen contents strengthen from 5.3% to 32.5% with increasing the oxidation time (Supplementary Table 2). The high-resolution O1s XPS spectra of micro/meso-PC-x (Fig. 5a–f) could be deconvoluted into three peaks located at 531.4 eV, 532.7 eV, and 534.0 eV, corresponding to carbonyl (-C = O), carboxyl (-COOH), and hydroxyl (-C-OH) surface functionalities, respectively^35^. The high-resolution C1s XPS spectra further reveals four peaks at 284.6 eV, 286.0 eV, 287.7 eV, and 289.4 eV, corresponding to the binding energy signals belonging to -C-C, -C-OH, -C = O, and -COOH groups, respectively (Supplementary Fig. 6a–f). Notably, the micro/meso-PC-x catalysts exhibit various quantities of oxygen functional groups (Supplementary Table 3), which are expected to fine-tune ORR activity for efficient H_2_O_2_ production.

**Fig. 6:**
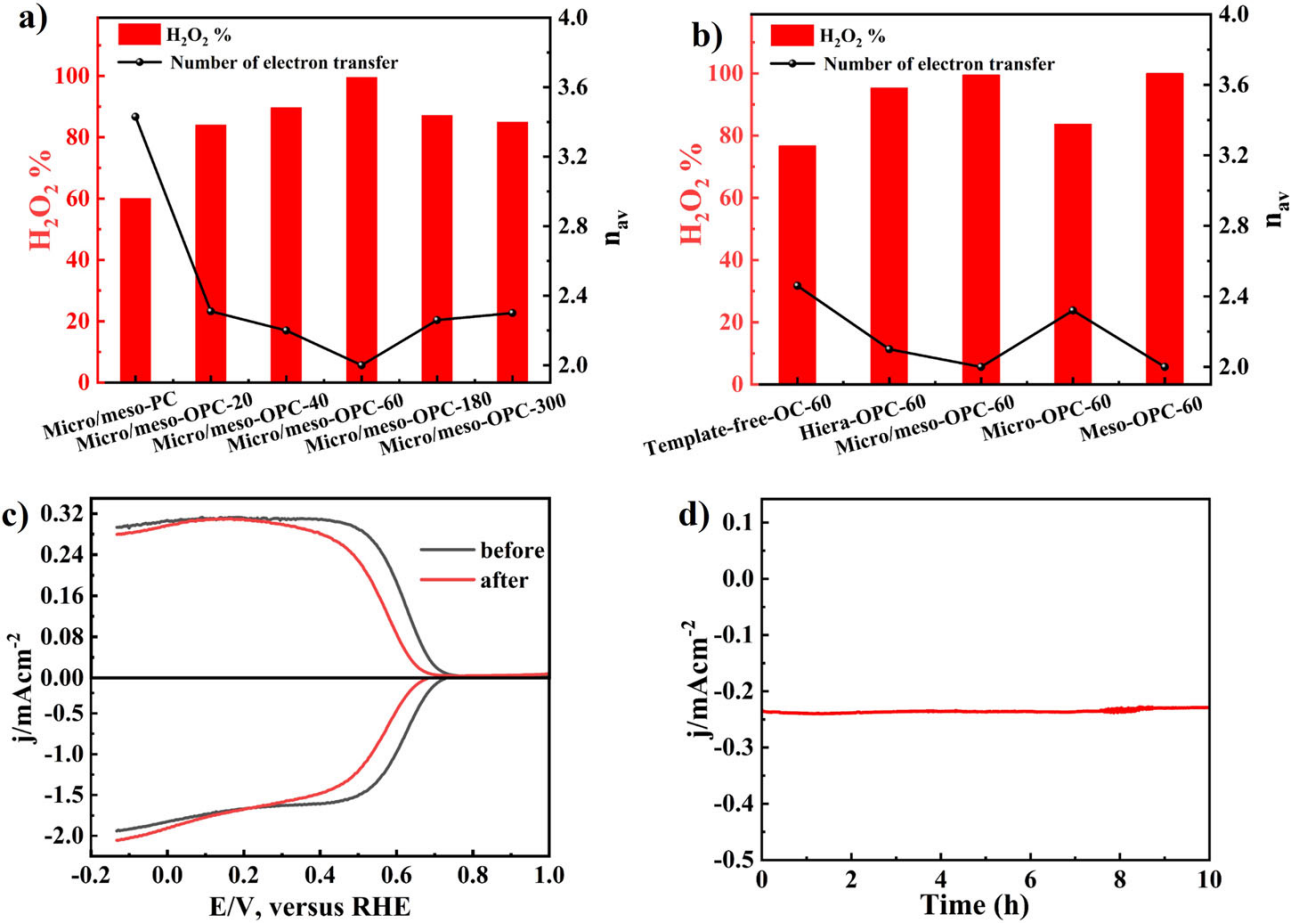
Electrochemical evaluation of catalysts. **a** Hydrogen peroxide selectivity (H_2_O_2_%) and average number of electron transferred (*n*_av_) of micro/meso-OPC-x in O_2_-saturated 0.1 M KOH solution, **b** hydrogen peroxide selectivity (H_2_O_2_%) and average number of electron transferred (*n*_av_) of Template-free-OC-60, Hiera-OPC-60, micro/meso-OPC-60, micro-OPC-60 and Meso-OPC-60 in O_2_-saturated 0.1 M KOH solution, **c** SCV-RRDE profiles of Meso-OPC-60 catalyst in an O_2_-saturated 0.1 M KOH electrolyte at 1600 rpm before and after reaction for 10 h. **d** Current density as function of reaction time at the constant potential of 0.2 V_RHE_ for long-term stability test of Meso-OPC-60 catalyst.

The H_2_O_2_ selectivity and the numbers of transferred electrons of the Micro/meso-OPC-x catalysts could be calculated from SCV-RRDE profiles as shown in Fig. 6a. The selectivity initially increases and subsequently decreases (from 60.0% to 95.5% and then 84.9%) with increased oxygen content, suggesting that moderate and proper surface content of oxygen is the key to the improved H_2_O_2_ electrochemical synthesis activity. The micro/meso-OPC-60 sample exhibits the best H_2_O_2_ selectivity among all micro/meso-OPC-x catalysts. The observed trend in H_2_O_2_ selectivity as a function of oxygen contents in micro/meso-OPC-x catalysts demonstrates a critical balance in surface oxygen functionalization for optimal electrocatalytic performance (Supplementary Fig. 7)^27,35^. This balance is further demonstrated by the remarkable selectivity of micro/meso-OPC-60, which can be attributed to the higher content of the -C = O group (Supplementary Fig. 8), highlighting its pivotal role as a vital active site in the electrochemical production of H_2_O_2_. The finding agrees well with previous research^36^ and provides additional experimental evidence that targeted functionalization can significantly contribute to superior catalytic performance. Notably, the decline in selectivity may arise from the excessive oxygen content within the catalyst structure, which may result in poor electron transportation^42,43^. In addition, over-high oxygen content may lead to strong interactions between oxygen-active sites and reaction intermediates, leading to the unwanted breaking of the O–O bond and thus the over-reduction yielding H_2_O^3^. Therefore, an in-depth understanding and precise control of oxygen content is essential for tailoring and optimizing the H_2_O_2_ selectivity in oxygen-functionalized carbon catalysts for efficient 2e^−^ ORR applications. To further elucidate the synergistic effect between the porous structure and oxygen function groups on H_2_O_2_ selectivity, we conducted a comprehensive analysis comparing catalysts featuring porous structure and oxygen functional groups and their integration. All porous materials and Template-free-C were oxidized in nitric acid for 60 min and subsequently analyzed via XPS, as depicted in Supplementary Fig. 9a–f and Supplementary Table 4. The results revealed distinct trend, the oxidized non-porous carbon (Template-free-OC-60) exhibited higher transferred electron numbers and the lowest H_2_O_2_ selectivity at 0.4 V_RHE_ in 0.1 M KOH solution as compared to its counterparts featuring both oxygen function groups and porous structure, irrespective of their specific porous structure (Fig. 6b). In particular, introducing oxygen to the non-porous carbon resulted in significant enhancement of H_2_O_2_ selectivity, from 28% to 76.7%. Furthermore, the construction of diverse porous structures within certain oxygen functionalities boosted selectivity to a range of 84.9–100%, emphasizing the significant roles of both factors in determining the catalytic activity. Mechanistic insights into this synergistic effect can be explained from a two-step promotional mechanism where the porous structure ensures efficient accessibility, mass transfer and product desorption^28^, while the oxygen functional groups act as catalytic active sites that preferentially drive the formation of H_2_O_2_ by altering the electronic structure of the adjacent carbon matrix^25,44^. This synergy between structural and functional groups highlights the essential of beneficial material design in optimizing the catalyst performance for specific reactions.

Following above structure-function relation guidance and combining the synergistic effect between porous structure and oxygen functionalities, we have fabricated Meso-OPC-60 carbon catalyst, which contains both mesopores and proper content of oxygen functionalities (Supplementary Table 3), and the optimized Meso-OPC-60 exhibits ~100% H_2_O_2_ selectivity at the potential of 0.4 *V*_RHE_, marking the highest recorded value compared to all the control samples in the present work or any other recently reported cutting-edge electrocatalysts^9,40,45,46^ (Supplementary Fig. 10). In addition, Meso-OPC-60 material also exhibits remarkable stability without significant change in ring and disk current densities after 10 h of continuous reaction (Fig. 6c, d), indicating that the catalyst has excellent durability.

## Conclusions

In conclusion, the present research has successfully synthesized various carbon materials with diverse porous structures and oxygen content. The mesoporous material (Meso-PC) displays outstanding 2e^−^ ORR selectivity, which can be attributed to its precisely tailored pore structure, facilitating the mass transfer of reactants and products. Furthermore, it is observed that the ketonic carbonyl (-C = O) group may be the main active site for 2e^−^ ORR and a moderate surface content of oxygen functionalities is the key to improved H_2_O_2_ electrochemical synthesis activity. Notably, the Meso-OPC-60 catalyst achieved 100% H_2_O_2_ selectivity at a potential of 0.4 *V*_RHE_. These findings provide a theoretical basis for improving the oxygen reduction performance of carbon nanomaterials for the highly efficient electrochemical synthesis of H_2_O_2_.

## Methods

### Synthesis of micro/mesoporous carbon (micro/meso-PC)

In a typical procedure for synthesizing micro/meso-PC, 1.25 g glucose was dispersed in 5 ml of purified water and stirred for approximately 30 min. Subsequently, a mixture of 1.0 g SBA-15, 1.0 g ZnCl_2_ and 0.14 g H_2_SO_4_ was added to the glucose solution and stirred for 6 h. The resulting product was placed to a Teflon-lined autoclave and kept at 100 °C for 6 h, followed by an additional 6 h at 160 °C. Afterward, the mixture was impregnated again with 5 ml H_2_O, 0.8 g glucose, and 0.14 g H_2_SO_4_, followed by heating at 100 °C for 6 h and 160 °C for 6 h. The obtained solid product was carbonized at 900 °C for 2 h under Ar flow. To remove the silica template, the SBA-15/carbon composite was dispersed in 5.0 M HF at room temperature, and the mixture was stirred for two days. The resulting suspension underwent centrifugation, raised with water, and dried at 60 °C, to obtain the micro/meso-PC.

### Synthesis of mesoporous carbon (meso-PC)

Meso-PC catalyst was synthesized using the same method described above, except for the absence of zinc chloride.

### Synthesis of microporous carbon (micro-PC)

Micro-PC catalyst was synthesized using the same method described above, except for the absence of zinc chloride and using Y-zeolite (SiO_2_/Al_2_O_3_) as a microporous template instead of mesoporous SBA-15.

### Synthesis of hierarchical porous carbon (hiera-PC)

Hiera-PC catalyst was synthesized by direct carbonization of the glucose and zinc chloride mixture with the mass ratio of 1:1 at 900 °C for 2 h under Ar flow.

### Synthesis of template-free carbon

Template-free-C catalyst was synthesized by direct carbonization of glucose at 900 °C for 2 h under Ar flow.

### HNO_3_-oxidation

200 mg above synthesized carbon materials were placed in a 100 mL one-neck flask containing 50 mL of 68% HNO_3_ and were refluxed at 120 °C. The suspension was centrifuged, rinsed with water, and dried at 60 °C to obtain micro/meso-OPC-x, where *x* represents the oxidation time, and *x* = 20, 40, 60, 180, and 300 min, respectively.

### Structure characterization

The morphological and microstructural properties of the catalyst materials were analyzed using a field emission scanning electron microscope (SEM) and a transmission electron microscope (TEM, FEI Tecnai G2 F20). The surface chemical compositions were quantified by X-ray photoelectron spectroscopy (XPS, ESCALAB250). Powder X-ray diffraction analysis was used to determine the crystallinity of the samples. The porosity of catalyst materials and pore size distribution were analyzed using the Brunauer-Emmett-Teller (BET) technique.

### Electrochemical measurements

The ORR studies were carried out at ambient temperature using a rotating ring disk electrode (RRDE) as the working electrode, a Pt wire as the counter electrode, and Hg/HgO (0.1 M KOH) as the reference electrode. The catalyst ink was obtained by combining 6 mg of the appropriate powder with 2 ml of a 0.3 wt% Nafion solution. The mixture was then subjected to ultrasonic treatment for 30 min. Next, 10 μL of the ink was deposited onto the working electrode. The SCV curves were obtained by employing a GC (glassy carbon) disk electrode surrounded by a Pt ring (with an inner diameter of 4 mm) in an O_2_-saturated solution of 0.1 M KOH. The scan rate was set at 10 mV/s, and the rotation rate was maintained at 1600 rpm. The H_2_O_2_ selectivity and number of electrons transferred were calculated using Eqs. (1) and (2), respectively.

1 $${{H}_{2}{O}_{2}} \% =2/(\sigma N+1)* 100 \%$$

2 $${n}_{{av}}=4\sigma N/(\sigma N+1)$$

where *σ* represents the ratio of the current densities of the disk (*j*D) and the ring (*j*R), and *N* is the collecting efficiency (*N* = 0.276).

### Supplementary information

Supplementary Information

Description of Additional Supplementary Files

Supplementary Data

## Data Availability

The data that support the findings of this study are available from the corresponding author upon reasonable request.
