# Supplementary material for: Effects of porous structure and oxygen functionalities on electrochemical synthesis of hydrogen peroxide on ordered mesoporous carbon
Source: Commun Chem. 2024 May 13;7:111. doi: 10.1038/s42004-024-01194-3 (PMC11091080; doi:10.1038/s42004-024-01194-3)
Supplement: Supplementary file 1 — Supplementary Information [file 42004_2024_1194_MOESM1_ESM.pdf]

Supporting information

Effects of porous structure and oxygen functionalities on electrochemical synthesis of hydrogen peroxide on ordered mesoporous carbon

Abdalazeez Ismail Mohamed Albashir [1, 2], Xingyu Lu[1, 2], Xueya Dai[1, 2], Wei Qi [1, 2] *

[1] *School of Materials Science and Engineering, University of Science and Technology of China, Shenyang 110016, Liaoning, People's Republic of China*

[2] *Shenyang National Laboratory for Materials Science, Institute of Metal Research, Chinese Academy of Sciences, Shenyang, Liaoning, People's Republic of China*

*Corresponding author: wqi@imr.ac.cn

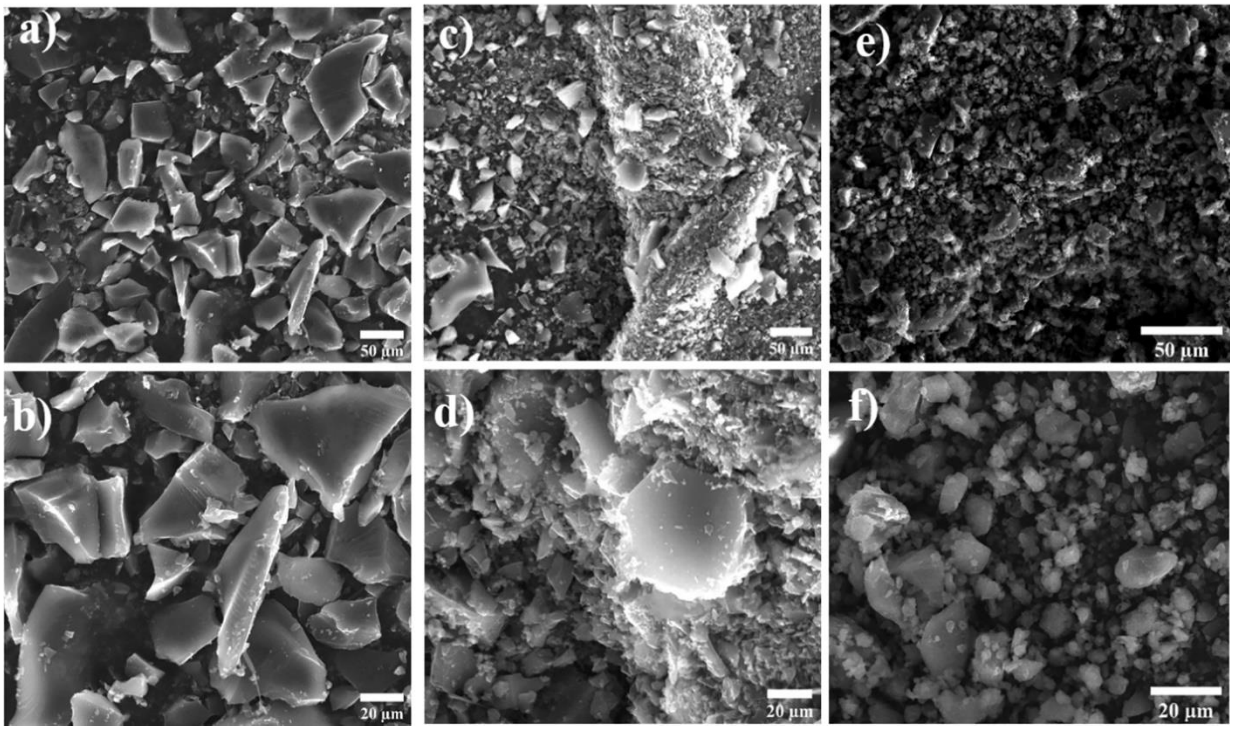

Supplementary Figure 1. Morphology characterizations of catalysts**.** SEM images of (a, b) Template-free-C, (c, d) of Hiera-PC and (e, f) of Micro/meso-PC.

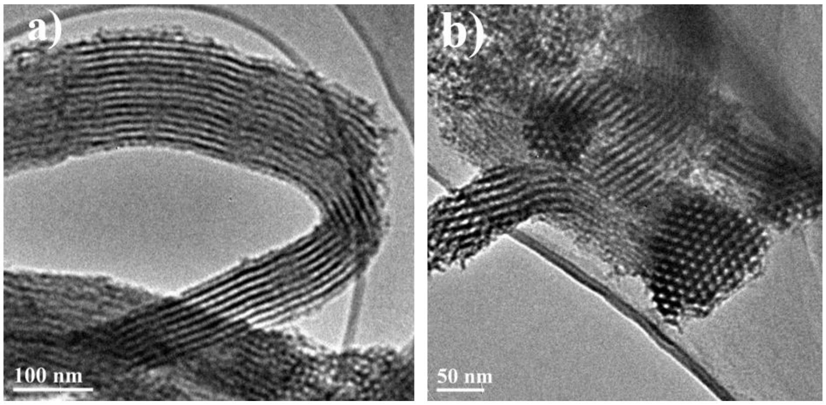

Supplementary Figure 2. Morphology characterizations of mesoporous silica-hard template (SBA-15). (a, b) TEM images of mesoporous silica-hard template (SBA-15) at different magnification.

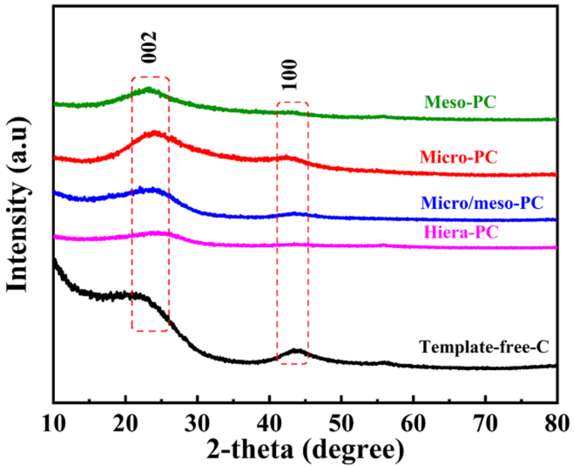

Supplementary Figure 3. XRD patterns of catalysts. X-ray diffraction (XRD) patterns of Template-free-C, Hiera-PC, Micro/meso-PC, Micro-PC and Meso-PC.

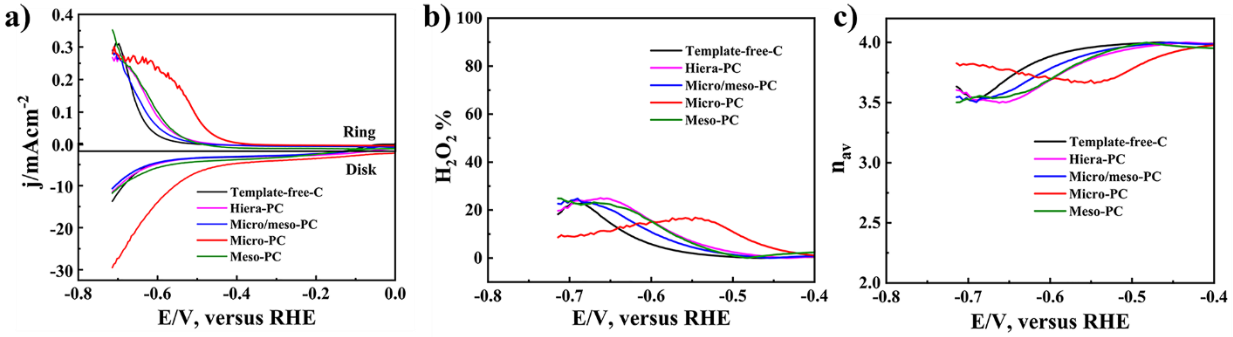

Supplementary Figure 4. Electrochemical evaluation in rotating ring-disk electrode (RRDE). a) SCV-RRDE profiles, b) Hydrogen peroxide selectivity (H_2O_2%), and c) Average number of electron transferred (n_{av}) of Template-free-C, Hiera-PC, Micro/meso-PC, Micro-PC and Meso-PC in O_2-saturated 0.1 M H_2SO_4 solution, respectively.

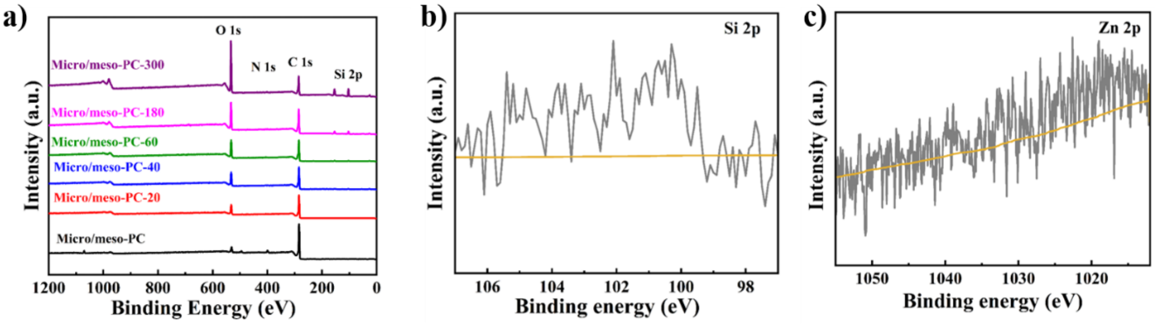

Supplementary Figure 5. XPS characterizations. a) XPS survey of Micro-OPC-x, b) XPS spectra of Si 2p and c) XPS spectra of Zn 2p.

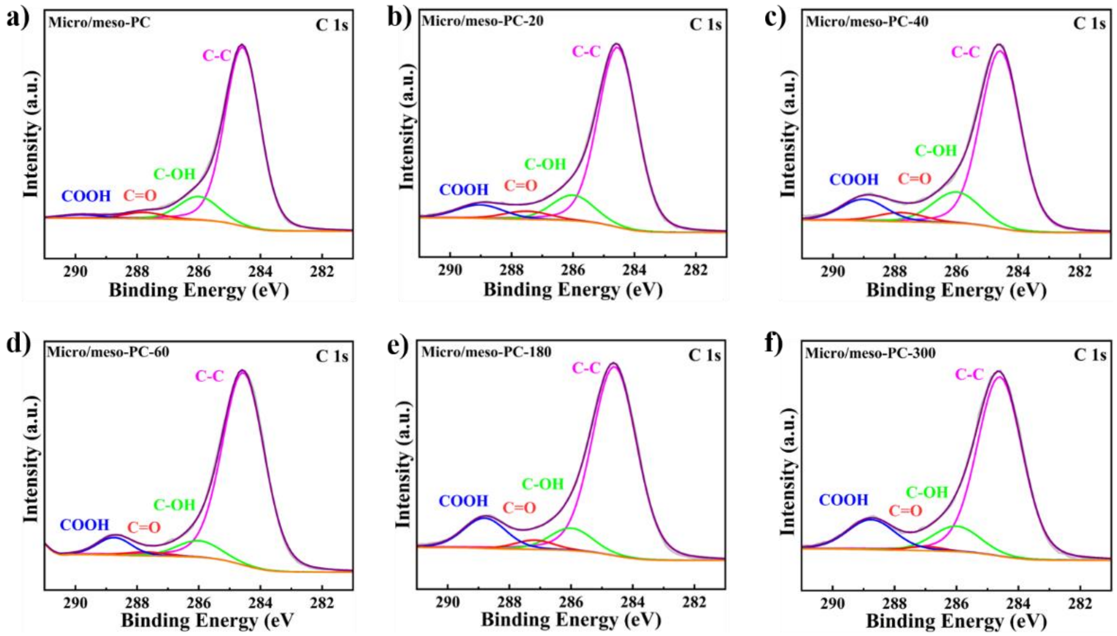

Supplementary Figure 6. Deconvoluted of C 1s XPS spectra. a) Micro/meso-PC, b) Micro/meso-OPC-20, c) Micro/meso-OPC-40, d) Micro/meso-OPC-60, e) Micro/meso-OPC-180 and f) Micro/meso-OPC-300, respectively.

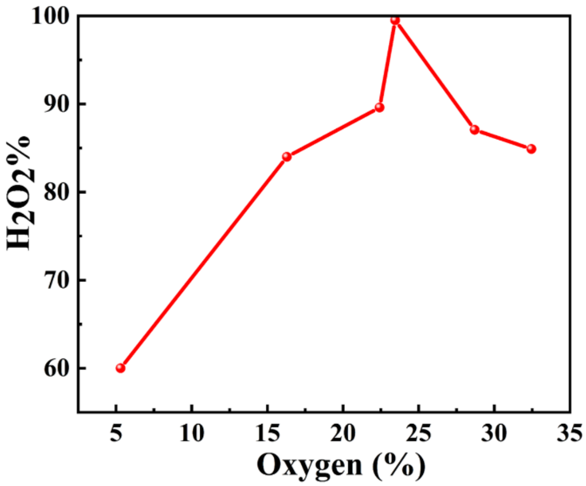

Supplementary Figure 7. Correlation between H_2O_2% and oxygen content. H_2O_2 selectivity as a function of the oxygen content.

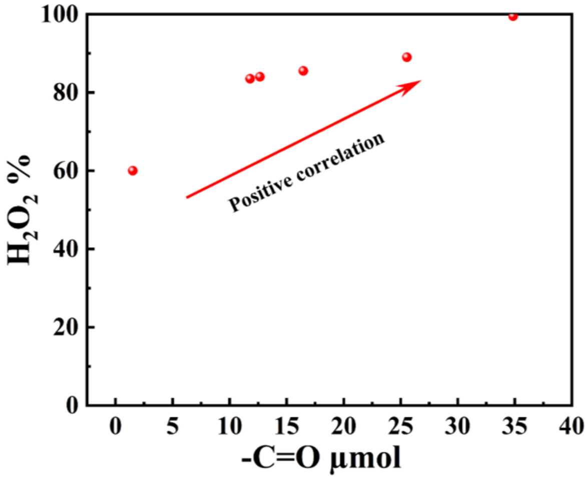

Supplementary Figure 8. Correlation between H_2O_2% and -C=O content. H_2O_2 selectivity as a function of the surface concentration of -C=O.

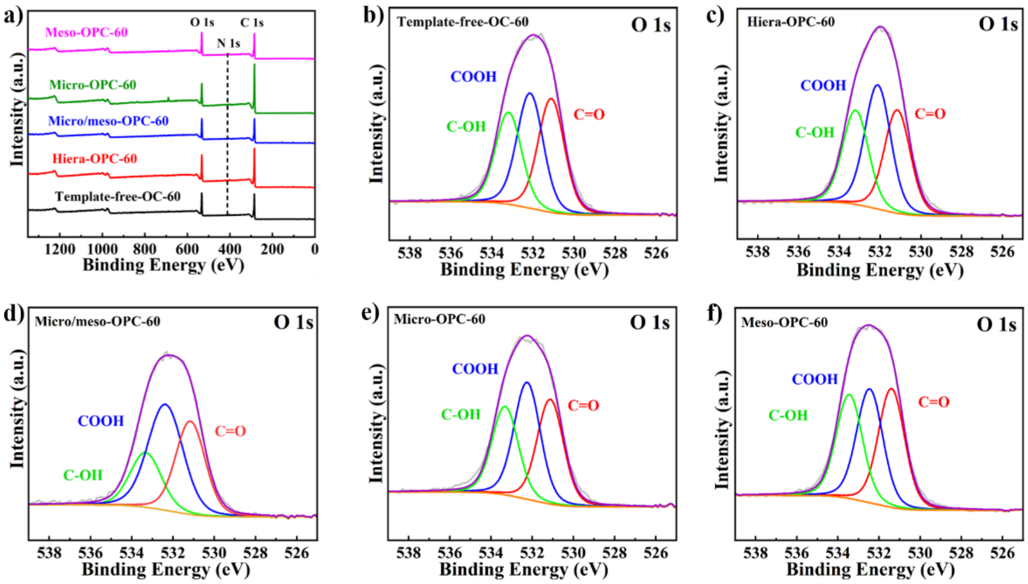

Supplementary Figure 9. XPS spectra. a) XPS survey of Template-free-OC-60, Hiera-OPC-60, Micro/meso-OPC-60, Micro-OPC-60 and Meso-OPC-60, O1s XPS spectra of b) Template-free-OC-60, c) Hiera-OPC-60, d) Micro/meso-OPC-60, e) Micro-OPC-60 and f) Meso-OPC-60, respectively.

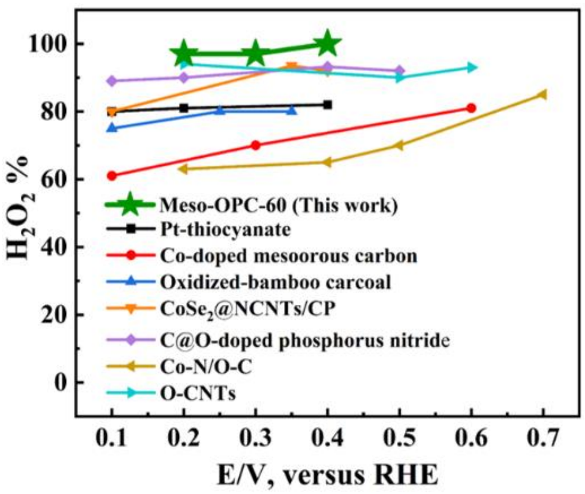

Supplementary Figure 10. Comparison of H_2O_2 selectivity with previous works. H_2O_2 % selectivity of Meso-OPC-60 compared with previously reported typical electrocatalysts for 2e$^-$ ORR.

Supplementary Table 1. Specific surface area and pore volume of Template-free-C, Hiera-PC, Micro/meso-PC, Micro-PC and Meso-PC.

sample	BET (m^2g^{-1})	V_t (cm^3g^{-1})	V_{micro} (cm^3g^{-1})
**Template-free**	169	0.069	0.055
**Hiera-P**C	1571	0.780	0.200
**Micro/meso-PC**	705	0.850	0.072
**Micro-PC**	858	0.498	0.215
**Meso-PC**	594	0.795	0.082

Supplementary Table 2. Contents of oxygen, nitrogen and carbon on the surface of Micro/meso-OPC-x.

**Sample**	**Oxygen (%)**	**Nitrogen (%)**	**Carbon (%)**
**Micro/meso-PC**	5.30	4.92	86.63
**Micro/meso-OPC-20**	16.29	1.51	82.20
**Micro/meso-OPC-40**	22.42	1.80	75.68
**Micro/meso-OPC-60**	23.45	3.29	72.29
**Micro/meso-OPC-180**	28.70	1.64	63.42
**Micro/meso-OPC-300**	32.45	1.33	60.22

Supplementary Table 3. Contents of oxygen, carboxylic group, carbonyl group and hydroxyl group on the surface of Micro/meso-OPC-x from O1s XPS measurements.

Sample	Oxygen (%)	COOH (%)	C=O (%)	C-OH (%)
**Micro/meso-PC**	5.3	2.26	1.24	1.78
**Micro/meso-OPC-20**	16.29	7.25	4.18	4.84
**Micro/meso-OPC-40**	22.42	8.5	6.36	7.55
**Micro/meso-OPC-60**	23.45	11.0	8.60	3.83
**Micro/meso-OPC-180**	28.7	24.44	2.83	1.42
**Micro/meso-OPC-300**	32.45	27.86	2.5	2.07

Supplementary Table 4. Contents of oxygen, nitrogen and carbon on the surface of Template-free-OC-60, Hiera-OPC-60, Micro/meso-OPC-60, Micro-OPC-60 and Meso-OPC-60.

Sample	Oxygen (%)	Nitrogen (%)	Carbon (%)
**Template-free-OPC-60**	19.84	1.43	78.72
**Hiera-OPC-60**	20.24	1.51	82.20
**Micro/meso-OPC-60**	23.45	3.29	75.68
**Micro -OPC-60**	16.61	1.72	81.67
**Meso-OPC-60**	24.60	1.92	73.17
