# Supplementary material for: Effects of porous structure and oxygen functionalities on electrochemical synthesis of hydrogen peroxide on ordered mesoporous carbon
Source: Commun Chem. 2024 May 13;7:111. doi: 10.1038/s42004-024-01194-3 (PMC11091080; doi:10.1038/s42004-024-01194-3)
Supplement: Supplementary file 2 — Description of Additional Supplementary Files [file 42004_2024_1194_MOESM2_ESM.pdf]

Description of Additional Supplementary Files .

File name- Supplementary Data

File description- All sources' data underlying the graphs presented in the main figures are provided and uploaded as supplementary data.
